# A model-based approach for a practical dosing strategy for the short, intensive treatment regimen for paediatric tuberculous meningitis

**DOI:** 10.3389/fphar.2023.1055329

**Published:** 2023-04-25

**Authors:** Roeland E. Wasmann, Tiziana Masini, Kerri Viney, Sabine Verkuijl, Annemieke Brands, Anneke C. Hesseling, Helen McIlleron, Paolo Denti, Kelly E. Dooley

**Affiliations:** ^1^ Division of Clinical Pharmacology, Department of Medicine, University of Cape Town, Cape Town, South Africa; ^2^ World Health Organization, Global Tuberculosis Programme, Geneva, Switzerland; ^3^ Desmond Tutu TB Centre, Department of Paediatrics and Child Health, Faculty of Medicine and Health Sciences, Stellenbosch University, Cape Town, South Africa; ^4^ Wellcome Centre for Infectious Diseases Research in Africa (CIDRI-Africa), Institute of Infectious Disease and Molecular Medicine, University of Cape Town, Cape Town, South Africa; ^5^ Division of Infectious Diseases, Vanderbilt University Medical Center, Nashville, TN, United States

**Keywords:** WHO, NONMEM, paediatric dosing, rifampicin, rifampin, isoniazid, pyrazinamide, ethionamide

## Abstract

Following infection with *Mycobacterium tuberculosis*, young children are at high risk of developing severe forms of tuberculosis (TB) disease, including TB meningitis (TBM), which is associated with significant morbidity and mortality. In 2022, the World Health Organization (WHO) conditionally recommended that a 6-month treatment regimen composed of higher doses of isoniazid (H) and rifampicin (R), with pyrazinamide (Z) and ethionamide (Eto) (6HRZEto), be used as an alternative to the standard 12-month regimen (2HRZ-Ethambutol/10HR) in children and adolescents with bacteriologically confirmed or clinically diagnosed TBM. This regimen has been used in South Africa since 1985, in a complex dosing scheme across weight bands using fixed-dose combinations (FDC) available locally at the time. This paper describes the methodology used to develop a new dosing strategy to facilitate implementation of the short TBM regimen based on newer globally available drug formulations. Several dosing options were simulated in a virtual representative population of children using population PK modelling. The exposure target was in line with the TBM regimen implemented in South Africa. The results were presented to a WHO convened expert meeting. Given the difficulty to achieve simple dosing using the globally available RH 75/50 mg FDC, the panel expressed the preference to target a slightly higher rifampicin exposure while keeping isoniazid exposures in line with those used in South Africa. This work informed the WHO operational handbook on the management of TB in children and adolescents, in which dosing strategies for children with TBM using the short TBM treatment regimen are provided.

## Introduction

Tuberculosis (TB) is a major global health problem with an estimated 9.9 million people falling ill and 1.5 million deaths, in 2020 (World Health Organization, 2021). Following infection with *Mycobacterium tuberculosis*, young children are particularly at risk of developing severe forms of TB, including TB meningitis (TBM), which is associated with significant morbidity and mortality (mortality estimated at 19.3%) and high rates (36.7%) of neurological sequelae among TBM survivors. Up to 15% of children with TB are diagnosed with TBM with an estimated 100000 cases per year. Given the severity of this form of TB, children are often hospitalized for diagnostic evaluation and treatment (Schaaf et al., 2007; Chiang et al., 2014; Wilkinson et al., 2017).

Early treatment with a combination of TB medicines and with doses aimed to optimize efficacy, can be life-saving and can potentially reduce neurological sequelae. However, data from randomized controlled clinical trials to inform optimal treatment regimens for TBM are lacking. Historically, the treatment of TBM in adults and children has used similar medicines as for the treatment of drug-susceptible pulmonary TB with the exception of replacing ethambutol with streptomycin and extending treatment to 12 months (World Health Organization, 2010a). In 2010, the World Health Organization (WHO) released a rapid advice communication recommending a regimen of 12 months duration for the treatment of TBM, consisting of isoniazid (H), rifampicin (R), pyrazinamide (Z), and ethambutol (E) for 2 months, followed by isoniazid and rifampicin for an additional 10 months (2HRZE/10HR); the recommended drug doses were the same as those for the treatment of pulmonary TB (i.e., HRZE 10/15/35/20 mg/kg daily) (World Health Organization, 2010b).

Varying penetration of first-line drugs through the blood-brain and the blood-cerebrospinal fluid (CSF) barriers in and a poor understanding of pharmacokinetics at the site of action (including the effect of protein binding) have historically made it difficult to determine the composition of the ideal regimen and dosing strategy to treat TBM. Indeed, while isoniazid and pyrazinamide show good CSF penetration, this is not true for ethambutol and rifampicin: ethambutol’s penetration into the CSF is poor, and rifampicin concentrations observed in the CSF are 5%–10% compared to those in plasma (Donald, 2010a; Donald, 2010b). For people with TBM, concerns about low concentrations of ethambutol and rifampicin at the site of disease have led clinicians to explore alternative options to ethambutol and the use of higher doses of rifampicin and isoniazid to achieve therapeutic concentrations in the brain and CSF (Ruslami et al., 2013). Some small trials have demonstrated benefits when using higher rifampicin dosages in adults and children with TBM (Ruslami et al., 2013; Savic et al., 2015; Svensson et al., 2020; Paradkar et al., 2022); larger, definitive trials are underway.

In South Africa, for several decades, paediatricians have been using a 6-month regimen with higher rifampicin and isoniazid doses (compared to standard WHO recommended dosing for the treatment of drug susceptible pulmonary TB) and replacing ethambutol with ethionamide (Eto) (6HRZEto) to treat TBM (Department of Health - Republic of South Africa, 2021). In observational cohorts, favourable treatment outcomes and no relapses have been observed with this regimen in a subset of patients who were followed up for 2 years after completing treatment (Donald et al., 1998). In one study, 95 children received the 6-month regimen, and although mortality was high (13 children died), the majority of children recovered. 14 In a subsequent study in 184 children, the mortality rate was 3.8% and although 5.6% of the children developed grade 3 or four adverse events related to elevated liver enzymes, all were able to restart the original regimen after liver enzymes normalised (van Toorn et al., 2014). This regimen, included in the South African national tuberculosis guidelines, is administered using daily doses of 20 mg/kg for isoniazid, rifampicin, and ethionamide, and 40 mg/kg for pyrazinamide.

A systematic review and meta-analysis was conducted to compare the effectiveness of the short, intensive regimen used in South Africa *versus* the WHO-recommended 12-month regimen to inform a WHO guideline update (Sulis et al., 2022). The authors report a treatment success (survival with or without sequelae) in 95% (95%CI 74%–99%) of the participants in the 6-month regimen *versus* 75% (95% CI: 69%–81%) in the 12-month regimen. Also in terms of neurological sequelae among survivors the 6-month regimen performed well with 36% (95% CI, 30%–43%) *versus* 66% (95% CI, 55%–75%) in the 12-month regimen (Sulis et al., 2022). Based on this, in March 2022, WHO recommended, that children and adolescents with bacteriologically confirmed or clinically diagnosed TBM (without suspicion or evidence of multi-drug resistant or rifampicin-resistant tuberculosis) could receive the 6-month intensive regimen (6HRZEto) as an alternative option to the 12-month regimen (2HRZE/10HR). (World Health Organization, 2022a).

In South Africa, the 6-month regimen had been administered using a dispersible fixed-dose combination (FDC) of RH 60/60 mg and additional 500-mg pyrazinamide and 250-mg ethionamide tablets as single-drug formulations. The dosing strategy for this regimen in the South African national guideline contains 14 weight bands between 3 and 25 kg, and some weight bands are dosed with quartered or halved tablets (Department of Health - Republic of South Africa, 2021). Moreover, the 1:1 isoniazid and rifampicin FDC used in South Africa is not globally available. Therefore, after the WHO recommendation was made, WHO aimed to develop a simple-to-implement dosing strategy that would achieve exposures in line with the South African 6-month regimen using globally available, child-friendly formulations.

Importantly, the dosing strategy aimed to achieve balanced exposure across weight bands. This acknowledges that administering the same mg/kg dose in all children can be detrimental, as it does not deliver similar exposures across children. It is known that the dose-exposure relationship across different weights and ages is non-linear due to the effects of allometry and maturation (Anderson and Holford, 2008; Denti et al., 2022a). This results in smaller children achieving lower exposures when receiving the same mg/kg dose, unless they are very young, with immature organ function, in which case the exposures may be higher (Denti et al., 2022a). Since TBM is highly fatal and causes significant morbidity in children, it is a condition for which precise dosing is especially critical.

To seek advice on the most suitable dosing strategy for the newly recommended 6-month intensive TBM regimen, WHO convened an expert consultation involving experts in clinical pharmacology, pharmacokinetics, and pharmacodynamics, as well as TB paediatricians and researchers. First, these experts reviewed the data collected in a meta-analysis to explore if 6HRZEto could be used (Sulis et al., 2022). After the advice that this regimen could indeed be recommended by the WHO, a dosing table needed to be created that took the globally available formulations into account. The process of the expert consultations was reported previously (World Health Organization, 2022b), while in this paper, we describe the methods and processes used to establish the new dosing guidance for the short, intensive TBM regimen, which involved running simulations incorporating established pharmacokinetic models. These simulations informed discussions around the best dosing strategy for the short, intensive TBM regimen, which led to the WHO guidance currently included in the operational handbook.

## Methods

### Pharmacokinetic models and Monte Carlo simulations

Our goal was to estimate plasma drug exposures that are achieved with different dosing strategies i.e., the original South African regimen with old 1:1 RH formulations and alternatives with currently available 3:2 RH formulations. We used previously published pharmacokinetic models of rifampicin, isoniazid, pyrazinamide, and ethionamide to simulate plasma drug exposures, i.e., steady-state area under the time-concentration curve over a 24-h dose interval (AUC_0-24_) (Nyberg et al., 2020; Denti et al., 2022b). These models include the effect of body size (either total body weight or fat-free mass) on clearance and volume of distribution (i.e., allometric scaling with fixed exponents of 0.75 and 1 on clearance and volume of distribution, respectively) and the effect of age on clearance (i.e., maturation) (Janmahasatian et al., 2005). The model for rifampicin also included the effect of age on bioavailability—whereby younger children have a lower bioavailability—and saturable hepatic elimination which helps describe the dose-exposure non-linearity seen with higher doses of rifampicin. The isoniazid model also included the effect of age on bioavailability and, additionally, the N-acetyltransferase 2 (NAT2) acetylator status to account for slow acetylating individuals having only half the clearance of fast acetylators (Denti et al., 2022b). Finally, the model of ethionamide included the drug-drug interaction of rifampicin on ethionamide clearance (Nyberg et al., 2020). All simulations were performed using NONMEM (version 7.4.2) and Perl-Speaks-NONMEM (version 4.8.8). R (version 4.1.2) was used for pre- and post-processing of data.

We performed Monte Carlo simulations using a representative virtual paediatric population of 160,000 children with a uniform weight distribution between 3 and 35 kg (5,000 children per 1-kg weight band), 50% female (Wasmann et al., 2021). This provided plausible and coherent values of age, weight, height, and sex that are covariates in the population PK models. The proportions of NAT2 acetylator status in the population, important for isoniazid pharmacokinetics, were set to 44% slow, 42% intermediate, and 14% fast, as reported in a study across eight high-burden countries (Gausi et al., 2021).

### Pharmacokinetic target

Currently, there is no evidence of a pharmacokinetic-pharmacodynamic target for paediatric TBM. With a lack of evidence, the AUC_0–24_ in steady-state conditions is the most robust index to compare exposures between two treatments. To evaluate whether the proposed dosing regimens achieve the desired exposure, we needed to define a target range within which the drug levels were deemed acceptable. As a first step, since no PK data were directly available from children receiving the South African 6-month regimen, we used population PK modelling and the original South African dosing guidelines to estimate the values of expected exposure levels. In addition to the original regimen, we also investigated rifampicin doses up to 30 mg/kg and isoniazid doses between 15 and 20 mg/kg. For each scenario, we translated the mg/kg dose to an exposure (AUC_0–24_) by using the median of the simulated exposures in the population over the whole weight range (i.e., these were the exposures that are expected in the South African 6-month regimen). These targets were presented to and discussed by an expert panel convened by WHO, who ultimately selected a target range for each drug.

### Dose selection

After selection of the target exposure range, we estimated the dose for each 1-kg weight band and each drug with population pharmacokinetic models, using a previously described method (Svensson et al., 2018). We developed an MS Excel dosing tool that would allow us and the expert panel to quickly visualise at a glance the approximate target attainment in each 1-kg weight band for each drug. The tool allows the user to select which formulations will be used, choosing amongst the five available child-friendly formulations and six adult formulations (shown in Table 1), and the number of tablets to administer in each specific weight range. With the tool, one can visualize how closely the selected dosing approaches will reach a dose resulting in an exposure within target exposures for each 1-kg weight band; additionally, there is a stratification for children under 3 months of age (with immature metabolism). Colour codes in the dosing tool indicate whether the exposure in the 1-kg band is expected to be within the range and, if not, how far (in percent) it is above or below the target. In the context of the advisory meeting, doses could be changed in the tool to explore different dosing strategies. Once final dosing was agreed upon, full simulations with the population pharmacokinetic models were performed to predict exact exposures.

**TABLE 1:** Globally available formulations at the time of dosing table development.

Child-friendly formulations	Adult formulations
**R/H 75/50 mg dispersible tablet^a^**	R/H 150/75 mg film-coated tablet
**Pyrazinamide 150 mg dispersible tablet^a^**	R/H 300/150 mg film-coated tablet
**Ethionamide 125 mg dispersible tablet^a^**	Isoniazid 300 mg uncoated tablet
R/H/Z 75/50/150 mg dispersible tablet	Pyrazinamide 400 mg uncoated tablet
Isoniazid 100 mg dispersible tablet	Pyrazinamide 500 mg uncoated tablet
	Ethionamide 250 mg uncoated tablet

a The formulations in bold were the preferential formulations for the dosing tables.

Abbreviations: R, rifampicin; H, isoniazid; Z, pyrazinamide.

When adjusting the dosing with the tool to select the most suitable regimen, several practical considerations were kept in mind. In general, narrow weight bands, and as such more complexity, are acceptable to attain a more precise dose given the serious nature of the disease. However, we aimed to minimize the number of weight bands to simplify dosing for a global audience. Children below 3 months of age have different pharmacokinetics due to immature organs and were considered as a separate group to improve their dose. To minimize drug manipulation, we aimed at implementing the dosing with whole tablets, whenever acceptable in terms of achieving the exposure target. When the use of half tablets was necessary to deliver the required exposure, this was allowed, especially since some of the formulations used have a functional scoring line or can be administered as aliquots after being dispersed in water. However, quartered tablets (used in the dosing strategy implemented in South Africa) were avoided as they would complicate administration and increase the chance of dosing errors. Wherever possible, we aimed to use FDCs to reduce tablet burden, in line with WHO’s recommendation to use FDCs over single-drug formulations for the treatment of people with drug-susceptible TB (World Health Organization, 2022c). However, the proposed dosing strategy is implemented only by using the RH 75/50 mg FDC, and not the RHZ 75/50/150 mg FDC, since the wrong ratio between HR and Z in the latter would have required additional top-ups of isoniazid and rifampicin, thus increasing tablet burden and requiring the availability and stocking of multiple formulations, some of which are not readily available in many settings. For similar reasons, we refrained from using standalone isoniazid formulations as a top-up.

Given the drug formulations that are widely available globally (Table 1), we designed a dosing strategy using child-friendly formulations for all children weighing between 3 and 35 kg. We also prepared two alternative approaches for children above 25 kg using adult formulations and either the 400 mg or the 500 mg pyrazinamide tablet.

## Results

**FIGURE 1:**
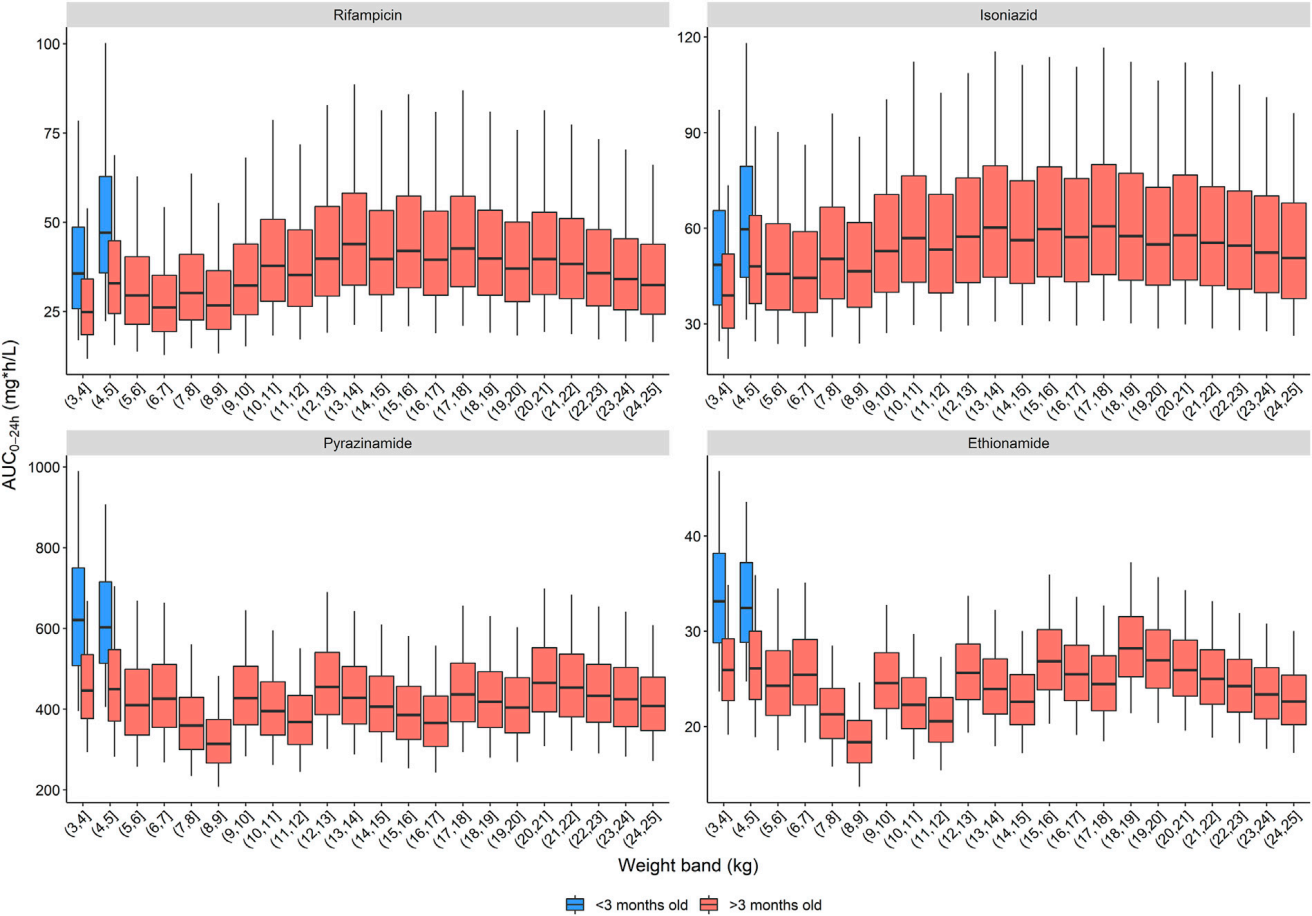
Area under the concentration-time curve over a 24-h dose interval (AUC_0–24h_) after dosing children according to the South African dosing table. The box represents the median and 25^th^ and 75^th^ percentile. The whiskers represent the 5^th^ and 95^th^ percentile.

The exposures predicted using the population pharmacokinetic models applied to historic South African dosing guidelines (RH in 1:1 formulation, weight bands as per 2011 guidance) are visualized in Figure 1 (Department of Health - Republic of South Africa, 2021). Overall, the expected median exposures of all four drugs are balanced over the whole weight range, except for children below 3 months of age. In these youngest children, exposures are higher than for older children of similar weights, and this is especially significant with pyrazinamide and ethionamide. The isoniazid exposure is highly variable due to the impact of NAT2 acetylator status.

Attaining exposures similar to those achieved historically with the RH 60/60 formulation using the newer globally available dispersible RH 75/50 mg FDC would require additional isoniazid top-ups using fractions of a tablet, a dosing strategy judged by the panel to be too complex and undesirable. As a compromise, one could increase the rifampicin target exposure and lower the isoniazid target exposure modestly. Exposures after doses up to 30 mg/kg rifampicin and down to 15 mg/kg isoniazid were simulated and presented to the expert panel. They approved of a compromise aiming at 22.5–30 mg/kg rifampicin dose while keeping the isoniazid dose at 15–20 mg/kg. For pyrazinamide and ethionamide, a target dose of 32–44 mg/kg and 16–22 mg/kg, respectively, were deemed acceptable by the panel.

For each of the recommended dose ranges, we simulated the exposure in children up to 25 kg and calculated the overall median AUC_0–24h_. The median exposures achieved at the lower and upper end of the dose range were used to define the target exposure range, e.g., from 22.5 to 30 mg/kg and 15 and 20 mg/kg for rifampicin and isoniazid, respectively. Exposure targets of 54.5–78.2, 47.6 to 59.4, 389 to 535, and 21.9–30.1 mg h/L were used for rifampicin, isoniazid, pyrazinamide, and ethionamide, respectively. The estimated doses per 1 kg weight band and per drug to get an exposure within the target range are included in the dosing tool, which has been added to the Supplementary materials.

**FIGURE 2:**
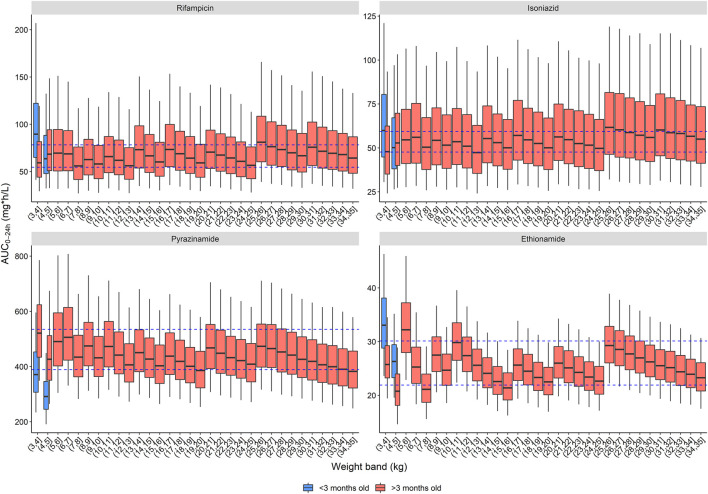
Area under the concentration-time curve over a 24-h dose interval (AUC_0–24h_) after the WHO recommended dose for children with child-friendly formulations. The box represents the median and 25^th^ and 75^th^ percentile. The whiskers represent the 5^th^ and 95^th^ percentile. The dashed blue lines represent the target ranges.

Three dosing strategies were recommended by the expert panel. The first, using only child-friendly formulations, is shown in Table 2 and Figure 2. We used three child-friendly formulations: the rifampicin/isoniazid 75/50 mg dispersible tablet, the 150 mg pyrazinamide dispersible tablet, and the 125 mg ethionamide dispersible tablet. A total of twelve weight bands were necessary for adequate dosing for the whole age and weight range, six of these for children below 10 kg. Although the median exposure in most 1 kg bands falls within the range, there were specific challenges in achieving exact targets for children below 3 months of age. Overall, the tablet burden using this approach is high, especially in children above 25 kg who will need to take 19 or more tablets daily.

**TABLE 2:** Dosing table using child-friendly formulations for children from 3 to 35 kg. The red and white horizontal bands in the formulation’s column represent the weight bands receiving the same dose. The colours in the last columns show if exposures are expected to be within (green) or outside the target range where yellow represents the percentage below the lower bound of the target range and purple represents the percentage above the higher bound of the target range. Abbreviations: *p*, child-friendly formulation; R, rifampicin; H, isoniazid; Z, pyrazinamide; Eto, ethionamide.

	Formulations				Simulated exposure				Difference from target range			
Weight band (kg)	pRH 75/50	pZ 150	pETO 125	Tablet burden	Rifampicin (mg·h/L)	Isoniazid (mg·h/L)	Pyrazinamide (mg·h/L)	Ethionamide (mg·h/L)	Rifampicin (%)	Isoniazid (%)	Pyrazinamide (%)	Ethionamide (%)
<3 months old												
3–4	1.5	0.5	0.5	4	89.6	59.9	371	33.1	14.6%	0.8%	−4.6%	9.8%
4–5	1.5	0.5	0.5	4	63.6	50.2	292	26.3	0.0%	0.0%	−24.9%	0.0%
>3 months old												
3–4	1.5	1	0.5	4	59.3	47.9	521	25.7	0.0%	0.0%	0.0%	0.0%
4–5	2	1	0.5	4	68.4	52.8	427	20.8	0.0%	0.0%	0.0%	−5.1%
5–6	2.5	1.5	1	6	69.5	54.6	491	32.2	0.0%	0.0%	0.0%	6.8%
6–7	3	2	1	6	68.7	56.1	506	25.3	0.0%	0.0%	0.0%	0.0%
7–8	3	2	1	6	56.0	50.4	434	21.1	0.0%	0.0%	0.0%	−3.7%
8–9	3.5	2.5	1.5	9	62.7	54.3	475	27.5	0.0%	0.0%	0.0%	0.0%
9–10	3.5	2.5	1.5	9	58.1	51.6	432	24.7	0.0%	0.0%	0.0%	0.0%
10–11	4	3	2	9	65.9	53.5	473	29.8	0.0%	0.0%	0.0%	0.0%
11–12	4	3	2	9	61.8	51.0	442	27.4	0.0%	0.0%	0.0%	0.0%
12–13	4	3	2	9	56.1	47.3	406	25.6	0.0%	−0.6%	0.0%	0.0%
13–14	5	3.5	2	11	73.2	55.3	450	24.1	0.0%	0.0%	0.0%	0.0%
14–15	5	3.5	2	11	66.7	53.0	427	22.6	0.0%	0.0%	0.0%	0.0%
15–16	5	3.5	2	11	60.2	50.1	403	21.4	0.0%	0.0%	0.0%	−2.4%
16–17	6	4	2.5	13	73.4	57.1	438	25.6	0.0%	0.0%	0.0%	0.0%
17–18	6	4	2.5	13	68.9	54.6	419	24.5	0.0%	0.0%	0.0%	0.0%
18–19	6	4	2.5	13	64.2	52.5	401	23.3	0.0%	0.0%	0.0%	0.0%
19–20	6	4	2.5	13	59.2	50.1	386	22.5	0.0%	0.0%	−0.7%	0.0%
20–21	7	5	3	15	70.6	56.3	468	26.0	0.0%	0.0%	0.0%	0.0%
21–22	7	5	3	15	67.5	54.7	448	25.1	0.0%	0.0%	0.0%	0.0%
22–23	7	5	3	15	64.4	52.5	432	24.3	0.0%	0.0%	0.0%	0.0%
23–24	7	5	3	15	60.8	51.6	422	23.4	0.0%	0.0%	0.0%	0.0%
24–25	7	5	3	15	56.6	49.7	409	22.6	0.0%	0.0%	0.0%	0.0%
25–26	9	6	4	19	81.1	61.7	473	29.3	3.7%	3.9%	0.0%	0.0%
26–27	9	6	4	19	76.3	60.3	465	28.6	0.0%	1.5%	0.0%	0.0%
27–28	9	6	4	19	73.3	58.8	453	27.7	0.0%	0.0%	0.0%	0.0%
28–29	9	6	4	19	69.9	57.2	442	27.0	0.0%	0.0%	0.0%	0.0%
29–30	9	6	4	19	66.8	56.0	426	26.2	0.0%	0.0%	0.0%	0.0%
30–31	10	6	4	20	75.8	60.2	419	25.5	0.0%	1.3%	0.0%	0.0%
31–32	10	6	4	20	71.5	58.7	406	25.2	0.0%	0.0%	0.0%	0.0%
32–33	10	6	4	20	69.3	58.2	400	24.4	0.0%	0.0%	0.0%	0.0%
33–34	10	6	4	20	68.0	56.7	390	23.9	0.0%	0.0%	0.0%	0.0%
34–35	10	6	4	20	64.3	55.0	383	23.3	0.0%	0.0%	−1.5%	0.0%
					66.9	54.6	430	25.2	0.0%	0.0%	0.0%	0.0%

The second and third dosing strategies employed adult formulations. Using adult tablets in children above 25 kg will lower pill burden, but the drug ratio of rifampicin and isoniazid is different (75/50 in child-friendly *versus* 150/75 in adult FDCs). As a result, there is no strategy possible where both rifampicin and isoniazid exposures are within the target range: either rifampicin exposure is above, or isoniazid exposure is below the target range. Since isoniazid dosing for TBM is already on the higher end of the doses commonly recommended to treat drug susceptible TB and given concerns about isoniazid’s related hepatotoxicity, the expert panel opted to have isoniazid exposures slightly below the target range. Two strategies using adult formulations are presented here: one using the 400 mg pyrazinamide adult tablet (Table 3) and one with the 500 mg adult tablet, given that both are available to national TB programmes (Table 4). The use of adult tablets in children above 25 kg can reduce the tablet burden to a maximum of 10 tablets per day.

**TABLE 3:** Dosing table using adult formulations for children from 25 to 35 kg and the 400 mg pyrazinamide formulation. The red and white horizontal bands in the formulation’s column represent the weight bands receiving the same dose. The colours in the last columns show if exposures are expected to be within (green) or outside the target range where yellow represents the percentage below the lower bound of the target range and purple represents the percentage above the higher bound of the target range. Abbreviations: a, adult formulation; R, rifampicin; H, isoniazid; Z, pyrazinamide; Eto, ethionamide.

	Formulations				Simulated exposure				Difference from target range			
Weight (kg)	aRH 150/75	aZ 400	aETO 250	Tablet burden	Rifampicin (mg·h/L)	Isoniazid (mg·h/L)	Pyrazinamide (mg·h/L)	Ethionamide (mg·h/L)	Rifampicin (%)	Isoniazid (%)	Pyrazinamide (%)	Ethionamide (%)
25–26	4	2	2	8	67.3	41.1	421	29.3	0.0	−13.7%	0.0%	0.0
26–27	4	2	2	8	63.4	40.2	413	28.6	0.0	−15.5%	0.0%	0.0
27–28	4	2	2	8	61.0	39.2	403	27.7	0.0	−17.6%	0.0%	0.0
28–29	4	2	2	8	58.2	38.1	393	27.0	0.0	−20.0%	0.0%	0.0
29–30	4	2	2	8	55.7	37.3	379	26.2	0.0	−21.6%	−2.5%	0.0
30–31	5	2	2	9	75.8	45.2	372	25.5	0.0	−5.0%	−4.3%	0.0
31–32	5	2	2	9	71.5	44.0	361	25.2	0.0	−7.6%	−7.2%	0.0
32–33	5	3	2	10	69.3	43.7	533	24.4	0.0	−8.2%	0.0%	0.0
33–34	5	3	2	10	68.0	42.5	521	23.9	0.0	−10.7%	0.0%	0.0
34–35	5	3	2	10	64.3	41.2	510	23.3	0.0	−13.4%	0.0%	0.0

**TABLE 4:** Dosing table using adult formulations for children from 25 to 35 kg and the 500 mg pyrazinamide formulation. The red and white horizontal bands in the formulation’s column represent the weight bands receiving the same dose. The colours in the last columns show if exposures are expected to be within (green) or outside the target range where yellow represents the percentage below the lower bound of the target range and purple represents the percentage above the higher bound of the target range. Abbreviations: a, adult formulation; R, rifampicin; H, isoniazid; Z, pyrazinamide; Eto, ethionamide.

	Formulations				Simulated exposure				Difference from target range			
Weight (kg)	aRH 150/75	aZ 500	aETO 250	Tablet burden	Rifampicin (mg·h/L)	Isoniazid (mg·h/L)	Pyrazinamide (mg·h/L)	Ethionamide (mg·h/L)	Rifampicin (%)	Isoniazid (%)	Pyrazinamide (%)	Ethionamide (%)
25–26	4	2	2	8	67.3	41.1	526	29.3	0.0	−13.7%	0.0	0.0
26–27	4	2	2	8	63.4	40.2	517	28.6	0.0	−15.5%	0.0	0.0
27–28	4	2	2	8	61.0	39.2	503	27.7	0.0	−17.6%	0.0	0.0
28–29	4	2	2	8	58.2	38.1	491	27.0	0.0	−20.0%	0.0	0.0
29–30	4	2	2	8	55.7	37.3	474	26.2	0.0	−21.6%	0.0	0.0
30–31	5	2	2	9	75.8	45.2	465	25.5	0.0	−5.0%	0.0	0.0
31–32	5	2	2	9	71.5	44.0	452	25.2	0.0	−7.6%	0.0	0.0
32–33	5	2	2	9	69.3	43.7	444	24.4	0.0	−8.2%	0.0	0.0
33–34	5	2	2	9	68.0	42.5	434	23.9	0.0	−10.7%	0.0	0.0
34–35	5	2	2	9	64.3	41.2	425	23.3	0.0	−13.4%	0.0	0.0

## Discussion

In this paper we describe how a practical dosing strategy for the treatment of paediatric TBM with the 6-month regimen using available child-friendly formulations was developed using population pharmacokinetic modelling and simulation. In the recommended dosing strategies, we aimed to find a compromise between close alignment with the target drug exposures *versus* practical considerations necessary for global implementation. Narrow weight bands and split tablets for children below 10 kg were necessary to avoid exposures outside the target range. For children above 10 kg, it was possible to employ a simpler approach and develop a dosing strategy with broader weight bands and, for the majority, use only whole tablets. children below 4 kg and younger than 3 months of age will receive a dose that will likely result in an exposure that is modestly above the selected target range for rifampicin, isoniazid and ethionamide and slightly below that for pyrazinamide. On the other hand, it is expected that these children will quickly grow and gain weight and move into higher weight bands rapidly. Moreover, these children would most likely be hospitalised and monitored for any possible toxicity.

The WHO convened a panel of global experts with expertise in clinical pharmacology, clinical management of paediatric TB, research, policy, community engagement, and programmatic implementation. All simulations were presented to the panel who considered efficacy, safety as well as implementation considerations (such as availability of drug formulations and feasibility of administration) before making their final dosing recommendations to WHO. The panel gave their recommendations in light of current evidence and practices regarding TBM, including the following considerations. i) TBM has high mortality, and post-treatment neurologic sequelae (which occur in about 53% of survivors) (Chiang et al., 2014). ii) The safety of a given drug may be different in ill, hospitalized children than in children receiving outpatient treatment for non-severe forms of TB, but hospitalised children are more closely monitored. iii) There are emerging data on the efficacy and safety of drugs in adult TBM that may be informative for paediatric TBM, especially for rifampicin, even at higher doses than currently recommended (Ruslami et al., 2013; Savic et al., 2015; Svensson et al., 2020). iv) We have good knowledge of the developmental pharmacology of drugs used in RHZEto and can use that information to achieve targets and reduce variability in drug exposures across children of different ages and weights. Final decisions regarding pharmacokinetic targets and dose selection were made by the panel.

In this study, we endeavoured to match the exposures children experienced when receiving the historic 6HRZEto regimen in South Africa. Whether or not those exposures are optimal for the treatment of paediatric TBM is unknown. Practically speaking, the use of the RH 75/50 mg FDC (rather than the RH 60/60 mg FDC) means that a scenario where both drugs are dosed at 20 mg/kg is no longer possible. As a compromise, in the recommended dosing strategies rifampicin exposure is higher while isoniazid is lower, compared to the historic South African dosing strategy. Some adult studies suggest even higher exposures may be beneficial, and a small trial among children suggests that neurocognitive outcomes may be improved with higher rifampicin dosing (Paradkar et al., 2022). A small, randomized trial in adult patients with TBM patients found that mortality decreased from 65% to 35% in patients in which the rifampicin dose was increased from 450 mg delivered orally to 600 mg given intravenously. The higher dose, given intravenously, resulted in a three-fold increase in rifampicin exposure (Ruslami et al., 2013). Furthermore, a model-based study showed that a 30 mg/kg oral rifampicin dose in children would be required for sufficient cerebrospinal fluid exposure and reduction in mortality (Savic et al., 2015). Finally, in a meta-analysis of randomized trials conducted among Indonesian adults with TBM, the exposure of rifampicin was strongly associated with reductions in mortality. Increasing the dose from 10 mg/kg to 30 mg/kg was predicted to increase 6-month survival from 50% to 70% (Svensson et al., 2020). In terms of safety, the data in children on higher dose rifampicin are limited. In adults, doses as high as 35–40 mg/kg appear to be well tolerated and safe (Boeree et al., 2017; Seijger et al., 2019). In children, a dose of 65–70 mg/kg was required to reach a similar exposure seen in adults on 35 mg/kg while a 50 mg/kg dose in these children given for a short time was well tolerated with no grade 3 or higher adverse events (Garcia-Prats et al., 2021). The data from adult trials are also informative when developing dosing strategies for children.

Treatment guidelines often recommend a mg per kg dose to treat people with TB. However, the relative weight range in children is large, for example, from 5 to 25 kg, there is a five-fold difference in body size and the allometric effect of body size on drug clearance significantly deviates from linearity in such a large range (Anderson and Holford, 2008). This means that when only considering body size, a child weighing 5 kg will receive a 5 times lower dose than a child weighing 25 kg, but drug clearance in that child is only 3.3 times lower (Denti et al., 2022a). When using an adult reference, the difference is even larger. As a result, children can be significantly underdosed when mg/kg dosing is used (Chabala et al., 2021; Garcia-Prats et al., 2021; Denti et al., 2022b). Furthermore, dosing children younger than 2 years of age is even more challenging because of the rapid maturation in organ function and fast growth, both of which impact drug clearance and thus exposure. Finally, metabolizing enzymes and drug excreting organs (i.e., kidneys) can mature at different rates and as a result the doses of some drugs need to increase rapidly when a child grows older while other drugs require a more gradual dose increase (Lu and Rosenbaum, 2014). It is therefore important that dose recommendations for children are no longer given as a mg per kg dose but include dosing tables and weight bands that are drug-specific.

The available FDCs are designed for the treatment of drug-susceptible TB using the standard doses recommended by WHO. The drug doses for rifampicin and isoniazid in the short TBM regimen are higher than those currently recommended for the treatment of drug-susceptible TB, and as a result there is a high tablet burden. Ideally, this would be solved by a formulation that contains higher doses of rifampicin and isoniazid. In practice, physicians could lower the tablet burden by using the child-friendly RHZ 75/50/150 mg FDC. However, this does make administration more complicated since this would require a “top-up” with the RH 75/50 mg FDC. For example, a 21 kg child could receive five RHZ 75/50/150 mg dispersible tablets, two RH 75/50 dispersible tablets and three 150 mg ethionamide dispersible tablets, reducing the tablet burden from 15 to 10.

An easy-to-use dosing tool was developed in MS Excel that enables users to design their own dosing strategy. The dosing tool bridges the gap between pharmacokinetic models and implementation considerations, to develop a dosing strategy for the short TBM regimen. The tool in the supplementary materials can also be used by physicians to look at alternative dosing strategies in case a recommended formulation is not available or if the tablet burden is high and needs to be lowered. However, it does have some inherent limitations. Because of the non-linear relationships between dose and exposure in young children (i.e., below 2 years of age) and saturable hepatic metabolism of rifampicin, the tool only provides an approximation of target attainment. For a more reliable exposure prediction we therefore simulated the dosing options in population models to confirm the appropriateness of the chosen dosing strategy. A better solution would be to have a browser-based platform (e.g., a Shiny application) that simulates exposures in the background while the user can focus on the formulations and weight bands.

The options from the process described in this paper were presented to a panel who then provided advice on a dosing strategy that enables the implementation of the short, intensive TBM regimen at country level, with globally available formulations.

## Conclusion

The development of dosing strategies should be informed by efficacy, safety, and pharmacokinetics data, as well as implementation considerations, such as availability of drug formulations that are appropriate for the specific target population. The combined effects of age and body size on drug exposures makes exposure predictions in children more complex than in adults, and straight mg/kg dosing that does not consider developmental pharmacology can result in toxic or subtherapeutic concentrations, especially for diseases in which precision dosing is essential. Here, we used a modelling approach to identify doses of the four drugs that comprise the newly WHO-recommended 6HRZEto regimen that achieve target exposures in children across the age and weight spectrum, taking into account available formulations, including widely used FDCs. Additionally, we provide a dosing tool that shows practitioners how to use either dispersible tablets or, for older children, adult tablets to achieve treatment goals. Young children are disproportionately at risk for TBM and its attendant high risk of morbidity and mortality. While the optimal doses for children remain to be established, we now have the tools to deliver a highly effective regimen to children globally (Sulis et al., 2022).

## Data Availability

The original contributions presented in the study are included in the article/Supplementary material, further inquiries can be directed to the corresponding author.
