# Supplementary material for: A model-based approach for a practical dosing strategy for the short, intensive treatment regimen for paediatric tuberculous meningitis
Source: Front Pharmacol. 2023 Apr 25;14:1055329. doi: 10.3389/fphar.2023.1055329 (PMC10167634; doi:10.3389/fphar.2023.1055329)
Supplement: Supplementary file 1 [file DataSheet1.PDF]

Title: A model-based approach for a practical dosing strategy for the short, intensive treatment regimen for paediatric tuberculous meningitis

Roeland E. Wasmann[1], Tiziana Masini[2], Kerri Viney[2], Sabine Verkuijl[2], Annemieke Brands[2], Anneke C. Hesseling[3], Helen McIlleron[1,4], Paolo Denti†[1], Kelly E. Dooley†[5]

[1] Division of Clinical Pharmacology, Department of Medicine, University of Cape Town, Cape Town, South Africa

[2] World Health Organization, Global Tuberculosis Programme, Geneva, Switzerland

[3] Desmond Tutu TB Centre, Department of Paediatrics and Child Health, Faculty of Medicine and Health Sciences, Stellenbosch University, Cape Town, South Africa

[4] Wellcome Centre for Infectious Diseases Research in Africa (CIDRI-Africa), Institute of Infectious Disease and Molecular Medicine, University of Cape Town, Cape Town, South Africa.

[5] Vanderbilt University Medical Center, Nashville, Tennessee, USA

† These authors contributed equally to this work and share last authorship

Corresponding author: Roeland E. Wasmann, PharmD, PhD

K45 Old Main Building, Groote Schuur Hospital, Observatory, Cape Town 7925 South Africa

roeland.wasmann@uct.ac.za, Phone number: +27 21 650 4861

Contents

Simulated exposures under the different recommendations 2

Model code for dose estimation per 1-kg weight band 5

Simulated exposures under the different recommendations

Table S1 Exposures (median [IQR]) when using the child-friendly formulations for children from 3 to 35 kg as shown in Table 2 in the manuscript.

Weight band (kg)	Rifampicin (mg·h/L)	Isoniazid (mg·h/L)	Pyrazinamide (mg·h/L)	Ethionamide (mg·h/L)
**<3 months old**				
**3-4**	89.6 (64.9 - 122)	59.9 (44.8 - 80.4)	371 (307 - 453)	33.1 (28.8 - 38.1)
**4-5**	63.6 (47.8 - 88.3)	50.2 (38.1 - 67.2)	292 (245 - 346)	26.3 (23.3 - 29.4)
**>3 months old**				
**3-4**	59.3 (44.1 - 81.6)	47.9 (35.1 - 62.4)	521 (434 - 624)	25.7 (23.2 - 29.2)
**4-5**	68.4 (50.9 - 94.2)	52.8 (40.0 - 69.7)	427 (351 - 512)	20.8 (18.1 - 24.0)
**5-6**	69.5 (50.6 - 94.5)	54.6 (41.1 - 72.0)	491 (402 - 594)	32.2 (27.9 - 37.2)
**6-7**	68.7 (50.8 - 93.3)	56.1 (41.3 - 75.4)	506 (423 - 614)	25.3 (22.2 - 29.0)
**7-8**	56.0 (41.5 - 76.0)	50.4 (37.7 - 67.2)	434 (363 - 513)	21.1 (18.7 - 24.0)
**8-9**	62.7 (46.4 - 84.1)	54.3 (40.3 - 72.8)	475 (403 - 560)	27.5 (24.4 - 30.9)
**9-10**	58.1 (42.4 - 77.6)	51.6 (38.4 - 68.8)	432 (364 - 509)	24.7 (21.8 - 27.7)
**10-11**	65.9 (48.8 - 87.0)	53.5 (40.5 - 72.4)	473 (399 - 563)	29.8 (26.5 - 33.5)
**11-12**	61.8 (46.6 - 83.3)	51.0 (38.6 - 68.9)	442 (373 - 525)	27.4 (24.4 - 30.8)
**12-13**	56.1 (41.8 - 75.3)	47.3 (35.5 - 62.8)	406 (344 - 483)	25.6 (22.7 - 28.6)
**13-14**	73.2 (53.7 - 98.3)	55.3 (41.5 - 73.9)	450 (381 - 533)	24.1 (21.4 - 27.0)
**14-15**	66.7 (49.0 - 89.2)	53.0 (39.7 - 69.3)	427 (361 - 504)	22.6 (20.2 - 25.4)
**15-16**	60.2 (45.0 - 81.0)	50.1 (37.8 - 66.2)	403 (339 - 478)	21.4 (19.1 - 24.2)
**16-17**	73.4 (55.1 - 99.8)	57.1 (43.1 - 77.1)	438 (371 - 522)	25.6 (22.7 - 28.6)
**17-18**	68.9 (51.3 - 92.6)	54.6 (41.1 - 72.6)	419 (353 - 496)	24.5 (21.9 - 27.6)
**18-19**	64.2 (48.0 - 87.0)	52.5 (39.8 - 70.4)	401 (341 - 470)	23.3 (20.9 - 26.2)
**19-20**	59.2 (43.9 - 78.8)	50.1 (37.7 - 67.0)	386 (323 - 455)	22.5 (20.1 - 25.2)
**20-21**	70.6 (53.2 - 93.8)	56.3 (42.8 - 75.0)	468 (392 - 552)	26.0 (23.2 - 29.1)
**21-22**	67.5 (50.3 - 89.9)	54.7 (41.0 - 72.0)	448 (376 - 532)	25.1 (22.3 - 28.3)
**22-23**	64.4 (47.7 - 86.5)	52.5 (39.3 - 71.1)	432 (369 - 510)	24.3 (21.7 - 27.2)

**23-24**	60.8 (44.5 - 81.3)	51.6 (38.3 - 69.1)	422 (358 - 498)	23.4 (20.9 - 26.2)
**24-25**	56.6 (42.4 - 76.1)	49.7 (37.1 - 66.2)	409 (344 - 483)	22.6 (20.2 - 25.4)
**25-26**	81.1 (60.3 - 108)	61.7 (46.3 - 81.5)	473 (398 - 554)	29.3 (26.0 - 32.8)
**26-27**	76.3 (56.7 - 103)	60.3 (44.7 - 81.0)	465 (396 - 552)	28.6 (25.5 - 32.0)
**27-28**	73.3 (55.3 - 99.0)	58.8 (44.3 - 78.5)	453 (380 - 536)	27.7 (24.8 - 31.0)
**28-29**	69.9 (51.7 - 94.0)	57.2 (43.1 - 76.4)	442 (372 - 524)	27.0 (24.1 - 30.1)
**29-30**	66.8 (49.5 - 90.4)	56.0 (42.3 - 74.2)	426 (360 - 508)	26.2 (23.4 - 29.4)
**30-31**	75.8 (56.4 - 102)	60.2 (45.5 - 80.8)	419 (351 - 497)	25.5 (22.9 - 28.5)
**31-32**	71.5 (53.6 - 97.1)	58.7 (44 - 78.5)	406 (343 - 481)	25.2 (22.5 - 28.2)
**32-33**	69.3 (52.2 - 93.2)	58.2 (43.2 - 77.0)	400 (337 - 476)	24.4 (21.8 - 27.4)
**33-34**	68.0 (50.5 - 90.4)	56.7 (42.5 - 74.4)	390 (330 - 464)	23.9 (21.4 - 26.7)
**34-35**	64.3 (48.2 - 86.7)	55.0 (41.3 - 73.5)	383 (322 - 456)	23.3 (20.8 - 26.1)
	66.9 (49.3 - 90.7)	54.6 (40.8 - 72.9)	430 (360 - 515)	25.2 (22.0 - 28.9)

Table S2 Exposures (median [IQR]) table using adult formulations for children from 25 to 35 kg and the 400 mg pyrazinamide formulation as shown in Table 3 in the manuscript.

**Weight band (kg)**	**Rifampicin (mg·h/L)**	**Isoniazid (mg·h/L)**	**Pyrazinamide (mg·h/L)**	**Ethionamide (mg·h/L)**
**25-26**	67.3 (50.1 – 90.0)	41.1 (30.9 - 54.3)	421 (354 - 492)	29.3 (26.0 - 32.8)
**26-27**	63.4 (47.1 - 85.2)	40.2 (29.8 – 54.0)	413 (352 - 491)	28.6 (25.5 – 32.0)
**27-28**	61.0 (46.0 - 82.3)	39.2 (29.5 - 52.3)	403 (338 - 477)	27.7 (24.8 – 31.0)
**28-29**	58.2 (43.1 - 78.3)	38.1 (28.8 - 50.9)	393 (331 - 466)	27.0 (24.1 - 30.1)
**29-30**	55.7 (41.3 - 75.3)	37.3 (28.2 - 49.5)	379 (320 - 451)	26.2 (23.4 - 29.4)
**30-31**	75.8 (56.4 - 102)	45.2 (34.1 - 60.6)	372 (312 - 442)	25.5 (22.9 - 28.5)
**31-32**	71.5 (53.6 - 97.1)	44.0 (33.0 - 58.9)	361 (305 - 428)	25.2 (22.5 - 28.2)
**32-33**	69.3 (52.2 - 93.2)	43.7 (32.4 - 57.7)	533 (449 - 635)	24.4 (21.8 - 27.4)
**33-34**	68.0 (50.5 - 90.4)	42.5 (31.9 - 55.8)	521 (440 - 619)	23.9 (21.4 - 26.7)
**34-35**	64.3 (48.2 - 86.7)	41.2 (30.9 - 55.1)	510 (430 - 608)	23.3 (20.8 - 26.1)

Table S3 Exposures (median [IQR]) table using adult formulations for children from 25 to 35 kg and the 500 mg pyrazinamide formulation as shown in Table 4 in the manuscript.

Weight band (kg)	Rifampicin (mg·h/L)	Isoniazid (mg·h/L)	Pyrazinamide (mg·h/L)	Ethionamide (mg·h/L)
**25-26**	67.3 (50.1 – 90.0)	41.1 (30.9 - 54.3)	526 (442 - 615)	29.3 (26.0 - 32.8)
**26-27**	63.4 (47.1 - 85.2)	40.2 (29.8 – 54.0)	517 (440 - 613)	28.6 (25.5 – 32.0)
**27-28**	61.0 (46.0 - 82.3)	39.2 (29.5 - 52.3)	503 (423 - 596)	27.7 (24.8 – 31.0)
**28-29**	58.2 (43.1 - 78.3)	38.1 (28.8 - 50.9)	491 (414 - 583)	27.0 (24.1 - 30.1)
**29-30**	55.7 (41.3 - 75.3)	37.3 (28.2 - 49.5)	474 (400 - 564)	26.2 (23.4 - 29.4)
**30-31**	75.8 (56.4 - 102)	45.2 (34.1 - 60.6)	465 (390 - 552)	25.5 (22.9 - 28.5)
**31-32**	71.5 (53.6 - 97.1)	44.0 (33.0 - 58.9)	452 (381 - 535)	25.2 (22.5 - 28.2)
**32-33**	69.3 (52.2 - 93.2)	43.7 (32.4 - 57.7)	444 (374 - 529)	24.4 (21.8 - 27.4)
**33-34**	68.0 (50.5 - 90.4)	42.5 (31.9 - 55.8)	434 (367 - 516)	23.9 (21.4 - 26.7)
**34-35**	64.3 (48.2 - 86.7)	41.2 (30.9 - 55.1)	425 (358 - 507)	23.3 (20.8 - 26.1)

Model code for dose estimation per 1-kg weight band

```
;; 1. Based on: run000

;; 2. Description: Dose estimation per 1-kg weight band for publication

;; x1. Author: Roeland Wasmann

; Settings for the memory of NONMEM

$SIZES MAXIDS=870548 NO=500 LTH=140

$PROBLEM ESTIMATION PER 1-KG WEIGHT BAND

$INPUT ID WT FFM SEX AGE SPEED DRUG DV CL V BIO KA MTT KM NN TIME AMT

$DATA data.csv IGNORE=@

; ID - subject identification number

; WT - Subject weight

; FFM - Subject FFM

; SEX - Subject Sex, 0 = female, 1 = male

; SPEED - NAT2 genotype for isoniazid (fast, intermediate, slow)

; DRUG - Flag to indicate to differentiate included drugs, here 1, 2, 3 and 4 for rifampicin, isoniazid,
pyrazinamide and ethionamide

; DV - Dependent variable to include in utility function, here set to zero (strive to minimize deviation ;
between target and individual exposure)

; CL - Individual apparent CL for each drug

; V  - Volume of distributionfor rifampicin only

; BIO - Individuals bioavailability for each drug

; KA - Individuals absorption rate constatnt for rifampicin only

; MTT - Individuals mean transit time for rifampicin only

; KM - Michaelis-Menten constant for rifampicin only

; NN - Number of transit compartment for rifampicin only

; TIME - Time where AUC is calculated for rifampicin only

; AMT - Amount of drug, not used since dose is estimated in code


$SUBROUTINE ADVAN13 TRANS1 TOL=9 ATOL=12

$MODEL     NCOMPARTMENTS=4 COMP=(ABS DEFDOSE) COMP=(LIVER)

        COMP=(CENTRAL) COMP=(AUC)
```

```
$PK
;--- Define doses and target AUCs for each drug
; Rifampicin
DINC0  = THETA(1)
DINC1  = THETA(2)
DINC2  = THETA(3)
DINC3  = THETA(4)
DINC4  = THETA(5)
DINC5  = THETA(6)
DINC6  = THETA(7)
DINC7  = THETA(8)
DINC8  = THETA(9)
DINC9  = THETA(10)
DINC10 = THETA(11)
DINC11 = THETA(12)
DINC12 = THETA(13)
DINC13 = THETA(14)
DINC14 = THETA(15)
DINC15 = THETA(16)
DINC16 = THETA(17)
DINC17 = THETA(18)
DINC18 = THETA(19)
DINC19 = THETA(20)
DINC20 = THETA(21)
DINC21 = THETA(22)
DINC22 = THETA(23)
DINC23 = THETA(24)
DINC24 = THETA(25)
DINC25 = THETA(26)
DINC26 = THETA(27)
```

```
DINC27 = THETA(28)
DINC28 = THETA(29)
DINC29 = THETA(30)
DINC30 = THETA(31)
DINC31 = THETA(32)
DINC32 = THETA(33)
DINC33 = THETA(34)

TARGET_L = 54.5          ; lower target range - median RIF AUC when giving 22.5 mg/kg
TARGET_H = 78.2          ; upper target range - median RIF AUC when giving 30mg/kg
ER = ETA(1)

; Isoniazid
IF(DRUG.EQ.2) THEN
        DINC0 = THETA(35)
        DINC1 = THETA(36)
        DINC2 = THETA(37)
        DINC3 = THETA(38)
        DINC4 = THETA(39)
        DINC5 = THETA(40)
        DINC6 = THETA(41)
        DINC7 = THETA(42)
        DINC8 = THETA(43)
        DINC9 = THETA(44)
        DINC10 = THETA(45)
        DINC11 = THETA(46)
        DINC12 = THETA(47)
        DINC13 = THETA(48)
        DINC14 = THETA(49)
```

```
    DINC15 = THETA(50)
    DINC16 = THETA(51)
    DINC17 = THETA(52)
    DINC18 = THETA(53)
    DINC19 = THETA(54)
    DINC20 = THETA(55)
    DINC21 = THETA(56)
    DINC22 = THETA(57)
    DINC23 = THETA(58)
    DINC24 = THETA(59)
    DINC25 = THETA(60)
    DINC26 = THETA(61)
    DINC27 = THETA(62)
    DINC28 = THETA(63)
    DINC29 = THETA(64)
    DINC30 = THETA(65)
    DINC31 = THETA(66)
    DINC32 = THETA(67)
    DINC33 = THETA(68)



    TARGET_L = 47.6          ; lower target range
    TARGET_H = 59.4          ; upper target range
    ER = ETA(2)
ENDIF

;Pyrazinamide
IF(DRUG.EQ.3) THEN
    DINC0 = THETA(69)
    DINC1 = THETA(70)
```

DINC2 = THETA(71)

DINC3 = THETA(72)

DINC4 = THETA(73)

DINC5 = THETA(74)

DINC6 = THETA(75)

DINC7 = THETA(76)

DINC8 = THETA(77)

DINC9 = THETA(78)

DINC10 = THETA(79)

DINC11 = THETA(80)

DINC12 = THETA(81)

DINC13 = THETA(82)

DINC14 = THETA(83)

DINC15 = THETA(84)

DINC16 = THETA(85)

DINC17 = THETA(86)

DINC18 = THETA(87)

DINC19 = THETA(88)

DINC20 = THETA(89)

DINC21 = THETA(90)

DINC22 = THETA(91)

DINC23 = THETA(92)

DINC24 = THETA(93)

DINC25 = THETA(94)

DINC26 = THETA(95)

DINC27 = THETA(96)

DINC28 = THETA(97)

DINC29 = THETA(98)

DINC30 = THETA(99)

DINC31 = THETA(100)

DINC32 = THETA(101)

```
        DINC33 = THETA(102)


        TARGET_L = 389              ; lower target range
        TARGET_H = 535              ; upper target range
        ER = ETA(3)
ENDIF

;Ethionamide
IF(DRUG.EQ.4) THEN
        DINC0 = THETA(103)
        DINC1 = THETA(104)
        DINC2 = THETA(105)
        DINC3 = THETA(106)
        DINC4 = THETA(107)
        DINC5 = THETA(108)
        DINC6 = THETA(109)
        DINC7 = THETA(110)
        DINC8 = THETA(111)
        DINC9 = THETA(112)
        DINC10 = THETA(113)
        DINC11 = THETA(114)
        DINC12 = THETA(115)
        DINC13 = THETA(116)
        DINC14 = THETA(117)
        DINC15 = THETA(118)
        DINC16 = THETA(119)
        DINC17 = THETA(120)
        DINC18 = THETA(121)
        DINC19 = THETA(122)
        DINC20 = THETA(123)
```

```
    DINC21 = THETA(124)
    DINC22 = THETA(125)
    DINC23 = THETA(126)
    DINC24 = THETA(127)
    DINC25 = THETA(128)
    DINC26 = THETA(129)
    DINC27 = THETA(130)
    DINC28 = THETA(131)
    DINC29 = THETA(132)
    DINC30 = THETA(133)
    DINC31 = THETA(134)
    DINC32 = THETA(135)
    DINC33 = THETA(136)

    TARGET_L = 21.9                 ; lower target range
    TARGET_H = 30.1                 ; upper target range
    ER = ETA(4)
ENDIF

;Weight bands
IF(AGE.LT.0.25.AND.WT.LT.4)                  DOSEFINAL = DINC0
IF(AGE.LT.0.25.AND.WT.GE.4.AND.WT.LT.5)      DOSEFINAL = DINC1
IF(AGE.GE.0.25.AND.WT.LT.4)                  DOSEFINAL = DINC2
IF(AGE.GE.0.25.AND.WT.GE.4.AND.WT.LT.5)      DOSEFINAL = DINC3
IF(WT.GE.5.AND.WT.LT.6)                      DOSEFINAL = DINC4
IF(WT.GE.6.AND.WT.LT.7)                      DOSEFINAL = DINC5
IF(WT.GE.7.AND.WT.LT.8)                      DOSEFINAL = DINC6
IF(WT.GE.8.AND.WT.LT.9)                      DOSEFINAL = DINC7
IF(WT.GE.9.AND.WT.LT.10)                     DOSEFINAL = DINC8
IF(WT.GE.10.AND.WT.LT.11)                    DOSEFINAL = DINC9
IF(WT.GE.11.AND.WT.LT.12)                    DOSEFINAL = DINC10
```

```
IF(WT.GE.12.AND.WT.LT.13)        DOSEFINAL = DINC11
IF(WT.GE.13.AND.WT.LT.14)        DOSEFINAL = DINC12
IF(WT.GE.14.AND.WT.LT.15)        DOSEFINAL = DINC13
IF(WT.GE.15.AND.WT.LT.16)        DOSEFINAL = DINC14
IF(WT.GE.16.AND.WT.LT.17)        DOSEFINAL = DINC15
IF(WT.GE.17.AND.WT.LT.18)        DOSEFINAL = DINC16
IF(WT.GE.18.AND.WT.LT.19)        DOSEFINAL = DINC17
IF(WT.GE.19.AND.WT.LT.20)        DOSEFINAL = DINC18
IF(WT.GE.20.AND.WT.LT.21)        DOSEFINAL = DINC19
IF(WT.GE.21.AND.WT.LT.22)        DOSEFINAL = DINC20
IF(WT.GE.22.AND.WT.LT.23)        DOSEFINAL = DINC21
IF(WT.GE.23.AND.WT.LT.24)        DOSEFINAL = DINC22
IF(WT.GE.24.AND.WT.LT.25)        DOSEFINAL = DINC23
IF(WT.GE.25.AND.WT.LT.26)        DOSEFINAL = DINC24
IF(WT.GE.26.AND.WT.LT.27)        DOSEFINAL = DINC25
IF(WT.GE.27.AND.WT.LT.28)        DOSEFINAL = DINC26
IF(WT.GE.28.AND.WT.LT.29)        DOSEFINAL = DINC27
IF(WT.GE.29.AND.WT.LT.30)        DOSEFINAL = DINC28
IF(WT.GE.30.AND.WT.LT.31)        DOSEFINAL = DINC29
IF(WT.GE.31.AND.WT.LT.32)        DOSEFINAL = DINC30
IF(WT.GE.32.AND.WT.LT.33)        DOSEFINAL = DINC31
IF(WT.GE.33.AND.WT.LT.34)        DOSEFINAL = DINC32
IF(WT.GE.34)                         DOSEFINAL = DINC33

;----------------------------------------------------------------------------------------------------------------------------
IF(DRUG.EQ.1) THEN
QH =90 * (FFM/56)**0.75
FU = 0.2
VH = 1 * (FFM/56)
CLINT = CL
LOGKM = KM ; LOG KM - claculated from data set - median of max conc in the liver
```

VMAX = CLINT*EXP(LOGKM) ; max enzimatic rate from eq. CLint = Vmax/KM

;

; Transit compartment absorption

F1=0 ; I need to set bioavailability in compartment 1 to 0 for this implementation of the transit compartment absorption

KTR = (NN+1)/MTT

IF (NEWIND/=2.OR.EVID>=3) THEN ; new individual, or reset event

    TNXD=TIME ; Time of the dose

    PNXD=DOSEFINAL ; Amount. If it's zero, the DE is deactivated.

ENDIF

TDOS=TNXD ; This will either save here the temporary values if it's a new individual...

PD=PNXD ; ...or the values which were read one record ahead during the execution of the previous record.

IF(AMT>0) THEN ; This reads one record ahead and stores the data to be used when running the following record

    TNXD=TIME

    PNXD=DOSEFINAL

ENDIF

; Uncomment this if you have ALAG or if you use ADDL

IF (DOSTIM>0) THEN ; This will account for the ADDL or lagged doses. It will overwrite the time, if it a non-event record

    TNXD=DOSTIM

    PNXD=DOSEFINAL

ENDIF

; To speed up the computation, I calculate here all the non-time-varying quantities used in $DES

PIZZA = LOG(BIO*PD*KTR + 0.00001) - GAMLN(NN+1)

```
ENDIF

; Initialisation
A_0(1) = 1E-12
A_0(2) = 1E-12
A_0(3) = 1E-12
A_0(4) = 1E-12

$DES
CH = A(2)/VH ; drug conc in liver

SAT_CL = 0
IF (CH>0) SAT_CL=VMAX/(1+EXP(-(LOG(CH)-LOGKM))) / (CH)

;transformation based on better Emax model, wider search in log parametrisation
EH  = (SAT_CL*FU)/((SAT_CL*FU)+QH) ; fraction undergoing first pass extraction
FH  = 1 - EH ;fraction available after 1st pass to go to systemic circulation

; COMPUTE RATE CONSTANTS
K20 = (QH*EH/VH)
K23 = (QH*FH/VH)
K32 = (QH/V)

TEMPO = T-TDOS ; this is time after dose for the transit, it should always be >= 0;

KTT = 0
TRANSIT = 0

IF(PD.GT.0.AND.TEMPO.GT.0) THEN ; This happens only id PD>0, so only if a dose has been detected
        KTT = KTR*(TEMPO)
```

```
        TRANSIT = EXP(PIZZA+NN*LOG(KTT)-KTT)
ENDIF
;
DADT(1) = TRANSIT -KA*A(1)
DADT(2) = KA*A(1) -K23*A(2) +K32*A(3) -K20*A(2)
DADT(3) = K23*A(2) -K32*A(3)

;-------------------------AUC AND CMAX----------------
CC = A(3)/V
TSS1=0
IF (T.GT.72)  TSS1=1
IF (T.GT.96)  TSS1=0
DADT(4) = CC*TSS1

$ERROR

;--- AUCs
IF(DRUG.EQ.1) THEN ;RIF
AUC = A(4)
ELSE
AUC = (DOSEFINAL*BIO)/CL
ENDIF

IF(AUC.LT.TARGET_L)                             DIFF = LOG(AUC)-LOG(TARGET_L)
IF(AUC.GT.TARGET_L.AND.AUC.LT.TARGET_H) DIFF = 0
IF(AUC.GT.TARGET_H)                             DIFF = LOG(AUC)-LOG(TARGET_H)

; DV is set to 0 so model looks for a dose that will result in a DIFF as close as possible to 0.
Y = DIFF + ER + EPS(1)

$THETA
```

(0, 112.5,1200) ; 1 RIF - 3 - 4 kg and <3mo

(0, 112.5,1200) ; 2 RIF - 4 - 5 kg and <3mo

(0, 112.5,1200) ; 3 RIF - 3 - 4 kg

(0, 150,1200)   ; 4 RIF - 4 - 5 kg

(0, 187.5,1200) ; 5 RIF - 5 - 6 kg

(0, 225,1200)   ; 6 RIF - 6 - 7 kg

(0, 262.5,1200) ; 7 RIF - 7 - 8 kg

(0, 262.5,1200) ; 8 RIF - 8 - 9 kg

(0, 300,1200)   ; 9 RIF - 9 - 10 kg

(0, 300,1200)   ; 10 RIF - 10 - 11 kg

(0, 337.5,1200) ; 11 RIF - 11 - 12 kg

(0, 337.5,1200) ; 12 RIF - 12 - 13 kg

(0, 375,1200)   ; 13 RIF - 13 - 14 kg

(0, 375,1200)   ; 14 RIF - 14 - 15 kg

(0, 450,1200)   ; 15 RIF - 15 - 16 kg

(0, 450,1200)   ; 16 RIF - 16 - 17 kg

(0, 450,1200)   ; 17 RIF - 17 - 18 kg

(0, 450,1200)   ; 18 RIF - 18 - 19 kg

(0, 525,1200)   ; 19 RIF - 19 - 20 kg

(0, 525,1200)   ; 20 RIF - 20 - 21 kg

(0, 525,1200)   ; 21 RIF - 21 - 22 kg

(0, 600,1200)   ; 22 RIF - 22 - 23 kg

(0, 600,1200)   ; 23 RIF - 23 - 24 kg

(0, 600,1200)   ; 24 RIF - 24 - 25 kg

(0, 600,1200)   ; 25 RIF - 25 - 26 kg

(0, 675,1200)   ; 26 RIF - 26 - 27 kg

(0, 675,1200)   ; 27 RIF - 27 - 28 kg

(0, 675,1200)   ; 28 RIF - 28 - 29 kg

(0, 675,1200)   ; 29 RIF - 29 - 30 kg

(0, 750,1200)   ; 30 RIF - 30 - 31 kg

(0, 750,1200)   ; 31 RIF - 31 - 32 kg

(0, 750,1200) ; 32 RIF - 32 - 33 kg

(0, 750,1200) ; 33 RIF - 33 - 34 kg

(0, 750,1200) ; 34 RIF - 34 - 35 kg

(0, 75,1200) ; 35 INH - 3 - 4 kg and <3mo

(0, 75,1200) ; 36 INH - 4 - 5 kg and <3mo

(0, 75,1200) ; 37 INH - 3 - 4 kg

(0, 100,1200) ; 38 INH - 4 - 5 kg

(0, 125,1200) ; 39 INH - 5 - 6 kg

(0, 150,1200) ; 40 INH - 6 - 7 kg

(0, 175,1200) ; 41 INH - 7 - 8 kg

(0, 175,1200) ; 42 INH - 8 - 9 kg

(0, 200,1200) ; 43 INH - 9 - 10 kg

(0, 200,1200) ; 44 INH - 10 - 11 kg

(0, 225,1200) ; 45 INH - 11 - 12 kg

(0, 225,1200) ; 46 INH - 12 - 13 kg

(0, 250,1200) ; 47 INH - 13 - 14 kg

(0, 250,1200) ; 48 INH - 14 - 15 kg

(0, 300,1200) ; 49 INH - 15 - 16 kg

(0, 300,1200) ; 50 INH - 16 - 17 kg

(0, 300,1200) ; 51 INH - 17 - 18 kg

(0, 300,1200) ; 52 INH - 18 - 19 kg

(0, 350,1200) ; 53 INH - 19 - 20 kg

(0, 350,1200) ; 54 INH - 20 - 21 kg

(0, 350,1200) ; 55 INH - 21 - 22 kg

(0, 400,1200) ; 56 INH - 22 - 23 kg

(0, 400,1200) ; 57 INH - 23 - 24 kg

(0, 400,1200) ; 58 INH - 24 - 25 kg

(0, 400,1200) ; 59 INH - 25 - 26 kg

(0, 450,1200) ; 60 INH - 26 - 27 kg

(0, 450,1200) ; 61 INH - 27 - 28 kg

(0, 450,1200) ; 62 INH - 28 - 29 kg

(0, 450,1200) ; 63 INH - 29 - 30 kg

(0, 500,1200) ; 64 INH - 30 - 31 kg

(0, 500,1200) ; 65 INH - 31 - 32 kg

(0, 500,1200) ; 66 INH - 32 - 33 kg

(0, 500,1200) ; 67 INH - 33 - 34 kg

(0, 500,1200) ; 68 INH - 34 - 35 kg

(0, 75,1200) ; 69 PZA - 3 - 4 kg and <3mo

(0, 75,1200) ; 70 PZA - 4 - 5 kg and <3mo

(0, 150,1200) ; 71 PZA - 3 - 4 kg

(0, 150,1200) ; 72 PZA - 4 - 5 kg

(0, 225,1200) ; 73 PZA - 5 - 6 kg

(0, 225,1200) ; 74 PZA - 6 - 7 kg

(0, 300,1200) ; 75 PZA - 7 - 8 kg

(0, 375,1200) ; 76 PZA - 8 - 9 kg

(0, 375,1200) ; 77 PZA - 9 - 10 kg

(0, 375,1200) ; 78 PZA - 10 - 11 kg

(0, 450,1200) ; 79 PZA - 11 - 12 kg

(0, 450,1200) ; 80 PZA - 12 - 13 kg

(0, 525,1200) ; 81 PZA - 13 - 14 kg

(0, 525,1200) ; 82 PZA - 14 - 15 kg

(0, 600,1200) ; 83 PZA - 15 - 16 kg

(0, 600,1200) ; 84 PZA - 16 - 17 kg

(0, 600,1200) ; 85 PZA - 17 - 18 kg

(0, 600,1200) ; 86 PZA - 18 - 19 kg

(0, 750,1200) ; 87 PZA - 19 - 20 kg

(0, 750,1200) ; 88 PZA - 20 - 21 kg

(0, 750,1200) ; 89 PZA - 21 - 22 kg

(0, 750,1200) ; 90 PZA - 22 - 23 kg

(0, 750,1200) ; 91 PZA - 23 - 24 kg

(0, 750,1200) ; 92 PZA - 24 - 25 kg

(0, 750,1200) ; 93 PZA - 25 - 26 kg

(0, 900,1200) ; 94 PZA - 26 - 27 kg

(0, 900,1200) ; 95 PZA - 27 - 28 kg

(0, 900,1200) ; 96 PZA - 28 - 29 kg

(0, 900,1200) ; 97 PZA - 29 - 30 kg

(0, 900,1200) ; 98 PZA - 30 - 31 kg

(0, 900,1200) ; 99 PZA - 31 - 32 kg

(0, 900,1200) ; 100 PZA - 32 - 33 kg

(0, 900,1200) ; 101 PZA - 33 - 34 kg

(0, 900,1200) ; 102 PZA - 34 - 35 kg

(0, 62.5,800) ; 103 ETO - 3 - 4 kg and <3mo

(0, 62.5,800) ; 104 ETO - 4 - 5 kg and <3mo

(0, 62.5,800) ; 105 ETO - 3 - 4 kg

(0, 62.5,800) ; 106 ETO - 4 - 5 kg

(0, 125,800) ; 107 ETO - 5 - 6 kg

(0, 125,800) ; 108 ETO - 6 - 7 kg

(0, 125,800) ; 109 ETO - 7 - 8 kg

(0, 187.5,800) ; 110 ETO - 8 - 9 kg

(0, 187.5,800) ; 111 ETO - 9 - 10 kg

(0, 187.5,800) ; 112 ETO - 10 - 11 kg

(0, 250,800) ; 113 ETO - 11 - 12 kg

(0, 250,800) ; 114 ETO - 12 - 13 kg

(0, 250,800) ; 115 ETO - 13 - 14 kg

(0, 250,800) ; 116 ETO - 14 - 15 kg

(0, 312.5,800) ; 117 ETO - 15 - 16 kg

(0, 312.5,800) ; 118 ETO - 16 - 17 kg

(0, 312.5,800) ; 119 ETO - 17 - 18 kg

(0, 312.5,800) ; 120 ETO - 18 - 19 kg

(0, 375,800) ; 121 ETO - 19 - 20 kg

```
(0, 375,800)     ; 122 ETO - 20 - 21 kg
(0, 375,800)     ; 123 ETO - 21 - 22 kg
(0, 375,800)     ; 124 ETO - 22 - 23 kg
(0, 375,800)     ; 125 ETO - 23 - 24 kg
(0, 375,800)     ; 126 ETO - 24 - 25 kg
(0, 375,800)     ; 127 ETO - 25 - 26 kg
(0, 437.5,800)   ; 128 ETO - 26 - 27 kg
(0, 437.5,800)   ; 129 ETO - 27 - 28 kg
(0, 437.5,800)   ; 130 ETO - 28 - 29 kg
(0, 437.5,800)   ; 131 ETO - 29 - 30 kg
(0, 500,800)     ; 132 ETO - 30 - 31 kg
(0, 500,800)     ; 133 ETO - 31 - 32 kg
(0, 500,800)     ; 134 ETO - 32 - 33 kg
(0, 500,800)     ; 135 ETO - 33 - 34 kg
(0, 500,800)     ; 136 ETO - 34 - 35 kg

$OMEGA
 0.01 FIX
 0.01 FIX
 0.01 FIX
 0.01 FIX

$SIGMA
 0.01 FIX

$ESTIMATION METHOD=0 MAX=9999 PRINT=5
$TABLE FILE=sdtab040 ID TIME WT FFM AGE SEX SPEED DRUG AUC DOSEFINAL NOPRINT NOAPPEND ONEHEADER
```
